# Impact of meteorological conditions and air pollution on COVID-19 pandemic transmission in Italy

**DOI:** 10.1038/s41598-020-73197-8

**Published:** 2020-10-01

**Authors:** Simone Lolli, Ying-Chieh Chen, Sheng-Hsiang Wang, Gemine Vivone

**Affiliations:** 1 CNR-IMAA, Contrada S. Loja S.N.C., 85050 Tito, PZ Italy; 2 Department of Atmospheric Sciences, National Central University, Taoyuan, 32001 Taiwan; 3 Center for Environmental Monitoring and Technology, National Central University, Taoyuan, 32001 Taiwan

**Keywords:** Environmental sciences, Environmental impact, Viral infection

## Abstract

Italy was the first, among all the European countries, to be strongly hit by the COVID-19 pandemic outbreak caused by the severe acute respiratory syndrome coronavirus 2 (Sars-CoV-2). The virus, proven to be very contagious, infected more than 9 million people worldwide (in June 2020). Nevertheless, it is not clear the role of air pollution and meteorological conditions on virus transmission. In this study, we quantitatively assessed how the meteorological and air quality parameters are correlated to the COVID-19 transmission in two large metropolitan areas in Northern Italy as Milan and Florence and in the autonomous province of Trento. Milan, capital of Lombardy region, it is considered the epicenter of the virus outbreak in Italy. Our main findings highlight that temperature and humidity related variables are negatively correlated to the virus transmission, whereas air pollution (PM_2.5_) shows a positive correlation (at lesser degree). In other words, COVID-19 pandemic transmission prefers dry and cool environmental conditions, as well as polluted air. For those reasons, the virus might easier spread in unfiltered air-conditioned indoor environments. Those results will be supporting decision makers to contain new possible outbreaks.

## Introduction

First cases of pneumonia of unknown origin were reported in Wuhan, Hubei Province, China late December 2019. Researchers identified the Severe Acute Respiratory Syndrome Coronavirus 2 (SARS-CoV-2) as the responsible of this disease in January 2020. The novel coronavirus shows a 70% genomic sequence similarity with SARS-CoV-1^1^. The first documented case is dated December, 31 2019, but the Chinese authorities track back cases up to November 2019. The disease is transmitted from human-to-human easily by close contact (in a radius of 1.5 m or more) through droplets, especially if an infected person is coughing, sneezing or just talking (at a lesser extent). It is still matter of debate if the virus can be transmitted by touching contaminated surfaces or its airborne permanence. A recent study^2^ put in evidence the natural origin of the virus, which seems to initiate from bats and infected humans through an interspecies spillover process that involves an intermediate host, e.g. snakes. The zoonotic virus can penetrate the human cellule through the ACE 2 receptor, and similarly to SARS, the incubation period (median) is 5.1 days (95% Confidence Interval, 4.5 to 5.8 days^3^). Anecdotal incubation period of 28 days is also reported. The infection is causing a wide range of symptoms and shows different degrees of severity with different fatality rates that are country dependent. Most common symptoms include dry cough, loss sense of smell, fever, tiredness. More serious symptoms include difficulty of breathing, shortness of breath, chest pain or pressure and loss of speech or movement. Usually, mild symptoms are managed at home, but critical ill patients need weeks of hospitalization in Intensive or Sub-Intensive Care Unit (ICU) with pulmonary ventilation. There is no specific anti-viral treatment or vaccine (June 2020) and the only effective preventive measure to block the virus outbreak is social distancing, limiting public gathering, especially indoor.

Studies on past coronavirus outbreaks put in evidence how the meteorological conditions are a co-factor in transmitting the virus transmission, enhancing or suppressing it. The atmospheric variables, i.e., ambient temperature and humidity, or the solar irradiation act differently with respect to the coronavirus survival, e.g.,^4,5^ show that the coronavirus transmission is facilitated in cold and dry weather. With respect to COVID-19 pandemic transmission, a recent Chinese study^6^ highlighted that the virus is favored with a temperature range between 5 and 15C. Another study assimilates temperature and relative humidity variables to improve the model prediction of infections and alert for a possible second pandemic outbreak^7^. Nevertheless, there is not still full agreement on how the meteorological variables influence the SARS CoV-2 virus transmission. Recently several studies investigated the contribution of meteorological conditions on COVID-19 transmission around the world, with different approaches. As shown in^8^, studies from China^9–12^, Iran, Spain, the USA^13^, Mexico^14^, Germany^15^, Turkey, Brazil, Indonesia, Norway and also over the globe^16^ are controversial and the World Health Organization (WHO) highlighted that new investigations are needed to quantitatively assess how the weather influence the virus spreading. On this topic^8^, results found that water vapor temperature, dew point, absolute and relative humidity show positive significant correlation with SARS CoV-19 transmission in Singapore, one of the biggest densely-populated megacities in South-East Asia.

On other hand, it seems that the SARS CoV-2 is selectively spreading, i.e. some large metropolitan areas are devastated by the virus in term of infected people and fatalities, while in others the virus transmission is limited with consequent much lesser fatalities. Air pollution is another crucial factor that should be taken into account to investigate the COVID-19 transmission and its role in leading to a more severe form of the disease. It has been formerly proven for SARS CoV-1 in 2002^17^ that air pollution can facilitate the virus transmission and increase its persistence in the atmosphere. In the United States, a study put in evidence that long-term exposure to high-concentration of particulate matter with an aerodynamic diameter less than 2.5 micron (PM_2.5_) increases the mortality^18^. In^19^ the authors investigated the aerodynamic nature of SARS CoV-2 sampling aerosols from different indoor environments. The results put in evidence that high concentrations of viral RNA are found in submicron aerosols, especially in Wuhan Hospital ICU rooms. However, the study cannot assess if the airborne aerosols carry a sufficient viral loading to infect people. Results from a recent study on 71 Italian provinces highlight that long-term air-quality data significantly correlated with cases of COVID-19. This result shows further evidence that chronic exposure to atmospheric contamination may represent a favorable context for virus spreading^20^. Lockdown policies introduced by local governments highlighted that the block of socio-economical activities improved air-quality^21^, not a sustainable solution in the long term^22^, but increased organic and inorganic waste that contaminated water^23^.

**Figure 1:**
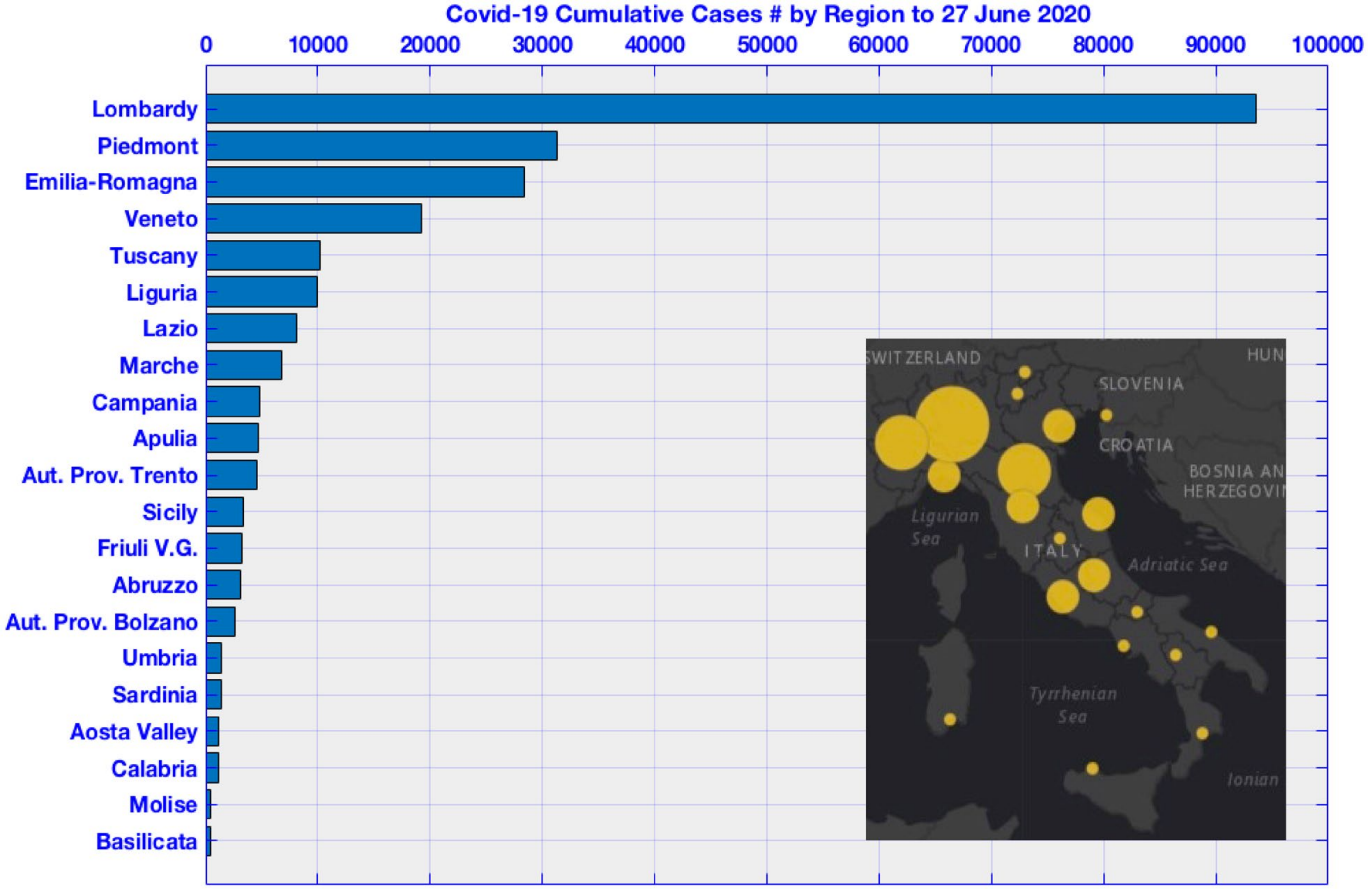
COVID-19 cumulative infected cases by region: COVID-19 pandemic transmission shows a transmission gradient between Northern and Southern regions. Lombardy, Veneto, Piedmont and Emilia-Romagna account for 60% of cases and 70% of deaths (as in June 2020). Inset: North–South gradient in COVID-19 pandemic transmission.

Italy is the first European and Western country heavily hit by the COVID-19 pandemic. As June 24th 2020, more than 239,000 cases are reported with about 34,000 fatalities. Almost 40% of documented cases and one third of fatalities are reported in Lombardy, the epicenter of the outbreak, a heavy industrialized and polluted region situated in the Po Valley (Fig. 1) that also experienced a significant drop in pollutant concentrations for PM_10_, PM_2.5_, NO_x_, SO_2_, CO and benzene^24^ during the lockdown. The map in Fig. 1 put in evidence how the Northern Italian regions are differently affected by the COVID-19 outbreak with respect to the Southern regions. Population density, even if playing a fundamental role in COVID-19 pandemic transmission, cannot be taken as an explicit evidence to explain the different transmission because other metropolitan areas in the southern regions show similar or higher population density, e.g. Naples.

The main scope of this manuscript is to investigate a possible correlation between meteorological parameters, air pollution and COVID-19 pandemic transmission over 103 days (8 March–19 June 2020) in two Italian metropolitan areas, i.e. Milan (Lombardy) and Florence (Tuscany) and the autonomous province of Trento. During the analyzed period, a strict lockdown was enforced by the Italian authorities. All the non-essential activities were shut down and the citizen mobility was minimized.

## Data and methodology

The proposed methodology tends to reproduce the analysis carried out in^8^ applied to the observations obtained in two metropolitan areas as Milan and Florence and the autonomous province of Trento. However, our approach is different from^8^, because we also considered other variables linked to the air pollution as the concentration of the Particulate Matter with an aerodynamic diameter less than 2.5 micron (PM_2.5_) and the Nitrogen Dioxide (NO_2_). Moreover, in the analysis we take into consideration the virus incubation period not considered in^8^. Instead of considering the number of daily positive cases as in^8^, we used as COVID-19 pandemic transmission outbreak variable, the number of the ICU daily patients (critical conditions) because this variable it is independent on the number of nasal swab performed tests and not subject to false positive/negative responses. Details are described in following sections.

### The metropolitan areas of Milan and Florence and the autonomous province of Trento

Milan (45.46 N, 9.19E, 52 m a.s.l) is a large metropolitan area, Lombardy business center, with a population of about 5 million. The region is heavily-industrialized, with the highest Italian Gross Domestic Product (GDP). Located in the Po Valley and surrounded by mountains, Alps to the North and Apennines to the South that inhibit wind circulation from sea and northern Europe, is also one of the most polluted hotspots in Europe, where the particular orography paired with aerosol emissions play a crucial role in deteriorating the air-quality^25^. The region is subject to a continental climate, experiencing humid hot summers and cold dry winters, where, especially during anticyclonic episodes, accomplice the lower planetary boundary layer height^26^, the city experiences higher atmospheric aerosol concentrations and persistent fog and haze. Similar meteorological and air pollution conditions are found in Milan neighbor cities, e.g. Bergamo and Brescia, also strongly hit by COVID-19 pandemic.

Florence, birthplace of Renaissance, is the capital of Tuscany and its metropolitan area hosts a population of 1.2 million. Yearly is visited by millions of tourists and it is one of the principal Italian attractions. It is located in the Center-North of Italy, about 300 km South–West of Milan, to which is daily connected by hundreds of fast speed trains. Florence is not affected by Po Valley pollution episodes, being shielded by the Apennines mountains on North–East side. Similar to Milan, but to a lesser degree, its metropolitan area experiences a continental climate, with cold winters and hot summers.

The autonomous province of Trento lies about 180 km North West from Milan towards Alps Mountains and hosts a population of about 0.6 million. The region, even under the influence of the Alpine/Sub-Alpine climate, depending on meteorological conditions, experiences pollution episodes that origins in the Po Valley.

### Data and estimations

The first reported official COVID-19 (not imported) case in Italy is recorded on 24 February 2020, about 60 km south of Milan. Regional and urban daily new infections, total ICU patients, cumulative fatalities and recovery are publicly available from the Italian civil protection department through GitHub (https://github.com/pcm-dpc/COVID-19).

To block the COVID-19 pandemic transmission, Italy put in place progressive and regional dependent population lockdown. In this study, we analyzed data from 8 March 2020 to 19 June 2020 (103 days). For the analysis, we considered the daily records of the most common meteorological parameters following the approach of^8^. The considered parameters with relative explanation are reported in Table 1.

**Table 1:** Basic Meteorological parameters obtained from Milano Linate airport observation site (https://www.wunderground.com/).

Parameters	Description (unit)
T_max_	Max temperature (°C)
T_avg_	Daily average temperature (°C)
T_min_	Min temperature (°C)
DP_max_	Max dew point (°C)
DP_avg_	Daily average dew point (°C)
DP_min_	Min dew point (°C)
RH_max_	Max relative humidity (%)
RH_avg_	Daily average relative humidity (%)
RH_min_	Min relative humidity (%)
WS_max_	Max horizontal wind speed (m s^−1^)
WS_avg_	Daily averaged horizontal wind speed (m s^−1^)
WS_min_	Min horizontal wind speed (m s^−1^)
P_max_	Max atmospheric pressure (hPa)
P_avg_	Daily average atmospheric pressure (hPa)
P_min_	Min atmospheric pressure (hPa)

The historical data are publicly available online (https://www.wunderground.com/). More information about data and data reliability can be found in^8^. Besides those variables, following again^8^ methodology, we retrieved also the absolute humidity (AH, in g m^−3^) through Clausius–Clapeyron equation^8^:

1 $$AH=2.1674\times RH\times \frac{6.112\times \mathrm{exp}\left(\frac{17.67\times T}{243.5+T}\right)}{273.15+T}$$

where *RH* represents the relative humidity and *T* the temperature. Following^8^, the water vapor (WV, in g kg^−1^) is estimated:

2 $$WV=6.22\times RH\times \frac{6.112\times \mathrm{exp}\left(\frac{17.67\times T}{243.5+T}\right)}{P}$$

where *P* is the atmospheric pressure.

Differently from^8^, to investigate a possible correlation with the air pollution, we also considered the PM_2.5_ and Nitrogen Dioxide (NO_2_) daily averaged concentrations. In Table 2 are reported the measurement stations with the relative lat/lon coordinates for Milan and Florence metropolitan areas and the autonomous province of Trento. Higher concentrations of PM_2.5_ and NO_2_ in the atmosphere have been already proven by previous study to be responsible of pulmonary diseases reducing life expectancy. PM_2.5_ and NO_2_ data are publicly available (upon request for Milan) at local Environmental Protection Agency (EPA) Websites. In Table 2 is also reported the position of the measurement stations used in this analysis and the different EPA websites.

**Table 2:** Location of the meteorological and air-quality stations.

	Met. parameters	Air quality	EPA website
Milan	Linate airport station 45.43N, 9.31E	Via Senato 45.46N, 9.19E	https://www.arpalombardia.it
Florence	Peretola airport 43.81°N, 11.18°E	Via Gramsci 43.77°N, 11.25°E	https://www.arpat.toscana.it/
Trento	Railway Station 46.07°N, 11.12°E	Parco S. Chiara 46.06°N, 11.12°E	https://bollettino.appa.tn.it/aria/

All the data, to reduce the measurement noise, are smoothed using a 5-day moving average window.

### Statistical approaches

Correlations between COVID-19 pandemic, meteorological variables and air pollution were investigated using non-linear Spearman and Kendall rank correlation tests, which have also employed in^6^. The Spearman rank correlation non-parametric test ${r}_{s}$ is described as follows:

3 $${r}_{s}=1-\frac{6\times {\sum }_{i}{d}_{i}^{2}}{n\left({n}^{2}-1\right)}$$

where ${d}_{i}$ represents the difference between the ranks of two parameters, and $n$ the number of alternatives. Equation 4 shows the Kendall rank correlation non-parametric test $\tau$:

4 $${\tau}=\frac{{{c}}{{o}}{{n}}{{c}}{{o}}{{r}}-{{d}}{{i}}{{s}}{{c}}{{o}}{{r}}}{0.5\times {{n}}\times \left({{n}}-1\right)}$$

Here $concor$ represents the number of concordant pairs, while $discor$ represents the discordant pairs, and $n$ is the number of pairs. A more detailed description of the statistical approaches can be found in^8^. Nevertheless, it is important to stress that values of ${r}_{s}$ and *τ* equal to + 1 and − 1 implying a perfect positive and negative correlation, respectively. The choice of these two non-parametric tests is based on the fact that the simpler linear correlations, e.g. Pearson, can’t be applied because the variables are not normally distributed, as shown from the statistical parameters, e.g. Kurtosis and asymmetry, of Tables 3, 5 and 7.

**Table 3:** Descriptive statistical analyses of meteorological and air-pollution variables (Feb. 19 – May 31, 2020; N = 103) in Milan, Lombardy.

Parameters	Min	Max	Mean	SD	Median	Mode	Kurtosis	Asymmetry
T_max_ (℃)	10	27	19	5	20	22	1.7	− 0.2
T_avg_ (℃)	7	22	14	5	15	9	1.6	0.0
T_min_ (℃)	1	17	8	5	7	3	1.5	0.2
DP_max_ (℃)	1	17	9	4	8	6	1.9	0.3
DP_avg_ (℃)	− 3	15	6	5	5	8	2.0	0.2
DP_min_ (℃)	− 9	14	3	6	3	− 1	2.3	0.2
P_max_ (hPa)	995	1016	1006	5	1007	1005	2.1	− 0.1
P_avg_ (hPa)	986	1013	1003	6	1004	1002	2.7	− 0.4
P_min_ (hPa)	985	1011	1001	6	1002	994	2.8	− 0.5
RH_max_ (%)	70	100	87	7	87	81	2.4	− 0.2
RH_avg_ (%)	39	84	62	10	62	56	2.5	− 0.1
RH_min_ (%)	14	65	40	11	40	33	3.1	− 0.1
AH_max_ (g m^−3^)	8	23	15	4	15	10	2.0	0.1
AH_avg_ (g m^−3^)	4	13	8	2	7	4	2.4	0.6
AH_min_ (g m^−3^)	1	8	4	2	4	1	2.7	0.7
WV_max_ (g kg^−1^)	6	20	12	3	13	6	2.0	0.1
WV_avg_ (g kg^−1^)	3	11	6	2	6	3	2.3	0.6
WV_min_ (g kg^−1^)	1	6	3	1	3	1	2.6	0.6
WS_max_ (m s^−1^)	9.0	21.6	16.0	2.9	16.0	16.0	2.3	− 0.3
WS_avg_ (m s^−1^)	4.5	10.8	8.0	1.5	8.3	8.3	2.6	− 0.4
WS_min_ (m s^−1^)	0.8	3.8	2.0	0.6	2.0	2.0	3.2	0.0
PM_2.5_ (µg m^−3^)	7.4	40.8	18.8	8.8	17.8	9.4	2.6	0.8
NO_2_ (ppb)	18.1	61.3	31.3	10.9	27.6	18.1	3.1	1.0

## Results and discussion

### Daily variation of COVID-19 cases, meteorological and air pollution variables

Differently from the approach proposed in^8^, where the correlation analysis was built upon the basis of COVID-19 new daily infections, we employed the residuals of the ICU patients with respect to a model. The daily new positives variable is highly chaotic and strictly correlated to the number of performed nasal test swabs, i.e. the more the test performed, the more positives are found. Moreover, delays in processing tests and false positives/negatives, being not uncommon and frequently reported, are factors that might introduce a bias in the analysis. For this reason, daily spikes in cases, without considering the incubation period, can be totally uncorrelated with the meteorological variables in^8^ for the reasons previously explained.

The number of hospitalized patients in ICU unit is a much stronger indicator of COVID-19 pandemic transmission, independent on the previously described sampling methods. We also considered, differently from^8^, the latency and the incubation period of the patients admitted into the ICU unit in critical conditions. From a recent study, the time to develop the Acute Respiratory Distress Syndrome (ARDS) from symptoms onset is 9 days^27^. Because ARDS requires ICU admission and 97% of the infected people develop symptoms after 11 days of incubation^3^, meteorological and air-pollution data are 20 days back time-shifted. This means that the daily number of ICU patients from 8 March 2020 to 19 June 2020 are the result of infections that happened from 18 February 2020 to 30 May 2020.

**Figure 2:**
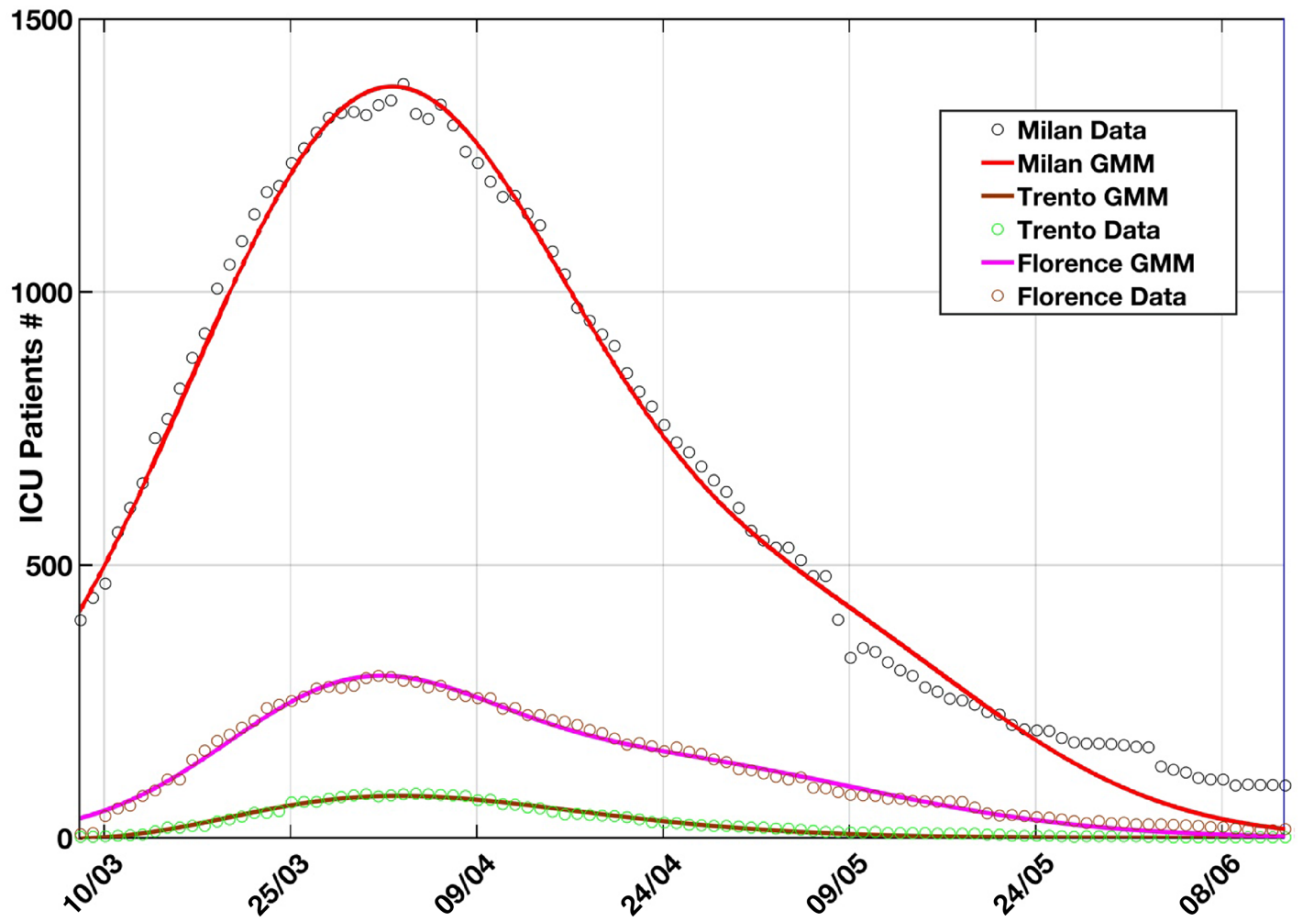
ICU Admitted patients fitted by a GMM^28^ extrapolated from the observed data in Milan, Florence and Trento. The residuals (Figs. 3, 4 and 5) are used to investigate the correlation with the meteorological and air-pollution variables.

The ICU daily cases are fitted with the Gaussian Mixture Model (GMM) defined in^28^ and applied to the COVID-19 pandemic variables. This approach is also followed in^29^. As it can be easily observed, the data show an early phase where the ICU patients grow exponentially, followed by reaching a peak and then an exponential drop. The curve symmetry is strictly dependent, among other variables, on lockdown adopted measures. Considering the residuals with respect to the observational data, i.e. the ICU patients number, makes the analysis independent on the analyzed period and lockdown policies, being the correlation analysis strongly dependent on the considered time period, i.e. the results from Spearman and Kendall rank tests performed during the early phase would give completely different results if the test were performed during the late phase. For these reasons, we correlate the meteorological and air-quality variables with respect to the ICU residual cases with respect to the GMM model, extrapolated from the data trend. The GMM model then accounts for the natural trend of viral epidemies and the effect of the lockdown on it. Thus, the residual analysis (i.e., the differences between the GMM and the observed cases) should preserve from spurious correlations between the above-mentioned effects and the parameters under analysis. Indeed, the considered atmospheric parameters quickly change (sometimes day-to-day), thus representing a divergence factor (residue) with respect to the model and characterizing the existing anomaly about the classical behavior described by the model. Figure 2 represents the GMM and the number of ICU patients.

#### Milan Metropolitan area

**Figure 3:**
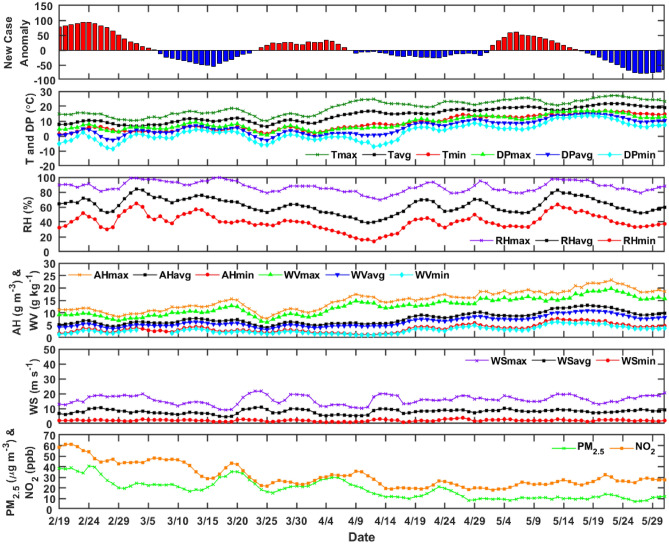
ICU patients daily case anomaly and meteorological and air pollution variables back time-shifted 20 days to take into consideration incubation and latency for Milan, Lombardy.

Figure 3 show the daily ICU patients anomaly with respect to the meteorological and air pollution variables for Milan. The daily variation of the meteorological and air-pollution parameters is also shown, together with a statistical analysis. In Table 3 it can be noticed that the temperature shows a large variation over the period, ranging from 1 °C to 27 °C. The dew point (DP) is the temperature to which air must be cooled to become saturated with water vapor is ranging between − 9 °C and 17 °C. Higher DP values (> 23 °C) are uncomfortable for humans and can induce heat stress^8^. The relative humidity, absolute humidity, and water vapor content are dependent variables, and range from 14 to 100% for RH, 1 to 23 g m^−3^ for AH, and 1 to 20 g Kg^−1^ for WV. The wind speed also shows a large variability, ranging from 0.8 to 21.6 m s^−1^. The air-pollution related parameters are affected by the lockdown restrictions. If considering the standard deviation, a temporal decrease is more evident in NO_2_ concentrations than PM_2.5_.

Table 4 shows the monthly variations of the basic meteorological variables and air pollution concentrations. As expected, the transition between winter to spring season shows an increase of both temperature and DP. Instead the RH remains constant within the standard deviation, AH shows a sharp increase during May, as the WV. The atmospheric pressure does not show a particular monthly variability like the horizontal wind speed. PM_2.5_ and NO_2_ concentrations, due to the block of human activity, show a substantial drop, more evident in NO_2_. We can speculate that the drop in NO_2_ is stronger because nitrogen dioxide is mainly produced by road traffic, while PM_2.5_ sources are road traffic, cooking and residence heating. After 40 days, NO_2_ is halved (Table 5).

**Table 4:** Meteorological parameters (mean ± SD) monthly variations in Milan, Italy for 2020.

	Feb. (N = 11)	March (N = 31)	April (N = 30)	May (N = 31)
T_avg_ (℃)	9 ± 1	9 ± 2	15 ± 3	20 ± 1
DP_avg_ (℃)	1 ± 3	3 ± 2	5 ± 4	12 ± 2
P_avg_ (hPa)	1004 ± 7	1003 ± 7	1004 ± 5	1003 ± 5
RH_avg_ (%)	63 ± 6	68 ± 8	55 ± 10	63 ± 10
AH_avg_ (g m^−3^)	6 ± 1	6 ± 1	7 ± 2	11 ± 2
WV_avg_ (g kg^−1^)	5 ± 1	5 ± 1	6 ± 1	9 ± 1
WS_avg_ (m s^−1^)	8.5 ± 1.6	7.6 ± 1.7	7.6 ± 1.6	8.5 ± 0.7
PM_2.5_ (µg m^−3^)	33.7 ± 6.9	23 ± 5.3	17.6 ± 6.7	14.5 ± 1.6
NO_2_ (ppb)	52.5 ± 7	36 ± 9.2	25.2 ± 5.5	25.1 ± 3.4

**Table 5:** Descriptive statistical analyses of meteorological and air-pollution variables (Feb. 19 – May 31, 2020; N = 103) in Florence, Tuscany.

Parameters	Min	Max	Mean	SD	Median	Mode	Kurtosis	Asymmetry
T_max_ (℃)	10	27	20	4	21	25	1.9	− 0.3
T_avg_ (℃)	7	21	14	4	14	16	1.8	0.1
T_min_ (℃)	2	16	8	4	6	4	1.8	0.4
DP_max_ (℃)	1	17	9	4	9	9	2.3	− 0.1
DP_avg_ (℃)	− 2	15	6	4	6	5	2.3	0.0
DP_min_ (℃)	− 6	13	3	5	3	0	2.3	0.2
P_max_ (hPa)	1004	1024	1015	5	1015	1017	2.1	0.1
P_avg_ (hPa)	905	1021	1008	16	1011	1007	25.6	− 4.5
P_min_ (hPa)	993	1018	1009	6	1009	1006	2.9	− 0.4
RH_max_ (%)	65	100	89	9	90	99	2.9	− 0.7
RH_avg_ (%)	37	82	63	11	64	59	2.4	− 0.4
RH_min_ (%)	10	62	39	12	42	46	2.4	− 0.5
AH_max_ (g m^−3^)	7	23	16	4	16	11	2.0	0.0
AH_avg_ (g m^−3^)	4	13	8	2	7	4	2.5	0.5
AH_min_ (g m^−3^)	1	7	4	2	3	1	2.6	0.5
WV_max_ (g kg^−1^)	5	19	13	3	14	5	2.0	0.0
WV_avg_ (g kg^−1^)	3	11	6	2	6	3	2.5	0.5
WV_min_ (g kg^−1^)	1	6	3	1	3	1	2.5	0.5
WS_max_ (m s^−1^)	11.5	32.6	20.9	3.6	21.2	22.6	4.2	− 0.1
WS_avg_ (m s^−1^)	5.2	17.3	9.2	2.1	9.2	7.5	6.1	1.1
WS_min_ (m s^−1^)	0.0	5.6	2.1	1.0	2.0	1.6	5.2	0.9
PM_2.5_ (µg m^−3^)	5.4	19.0	11.4	3.5	11.4	8.4	2.2	0.2
NO_2_ (ppb)	33.0	117.8	68.5	22.7	65.0	48.8	2.3	0.6

#### Florence Metropolitan area

**Figure 4:**
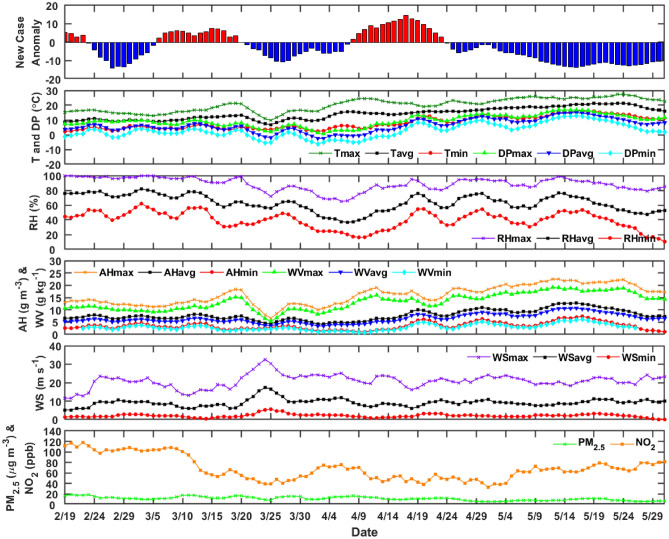
ICU patients daily case anomaly and meteorological and air pollution variables back time-shifted 20 days to take into consideration incubation and latency for Florence, Tuscany.

Similarly, for Florence metropolitan area, top of Fig. 4 shows the ICU cases anomaly together with the meteorological and air-pollution variables. The temperature ranges from 2 °C to 27 °C, while the DP from − 6 °C to 17 °C degrees. The humidity ranges from 10 to 100%, while the absolute humidity from 1 g m^−3^ to 23 g m^−3^. The water vapor concentration from 1 to 19 g kg^−1^. Those variables show a comparable variability with Milan.

The wind speed shows a larger variability with respect to Milan, 0–32.6 m s^−1^. The PM_2.5_ and NO_2_, also in Florence, because of lock-down, show a sensible reduction, as shown in Fig. 4 and Table 6. As expected, both temperature and humidity related parameters increase from February to May, while again the pressure is constant with respect to the Standard Deviation.

**Table 6:** Meteorological parameters (mean ± SD) monthly variations in Florence, Italy for 2020.

	Feb. (N = 11)	March (N = 31)	April (N = 30)	May (N = 31)
T_avg_ (℃)	10 ± 1	10 ± 2	15 ± 2	19 ± 1
DP_avg_ (℃)	5 ± 1	4 ± 2	5 ± 5	11 ± 3
P_avg_ (hPa)	1012 ± 6	1011 ± 6	1012 ± 4	1001 ± 27
RH_avg_ (%)	75 ± 3	68 ± 8	56 ± 13	61 ± 9
AH_avg_ (g m^−3^)	7 ± 1	7 ± 1	7 ± 2	10 ± 2
WV_avg_ (g kg^−1^)	6 ± 0	5 ± 1	6 ± 2	8 ± 1
WS_avg_ (m s^−1^)	7.9 ± 2.1	9.5 ± 3	9.1 ± 1.5	9.3 ± 1.1
PM_2.5_ (µg m^−3^)	15.5 ± 2.8	13.4 ± 2.7	11.4 ± 2.7	8.1 ± 1.9
NO_2_ (ppb)	107.7 ± 6.4	71.2 ± 26	54.7 ± 11.4	65.2 ± 12.7

#### Trento autonomous Province

**Figure 5:**
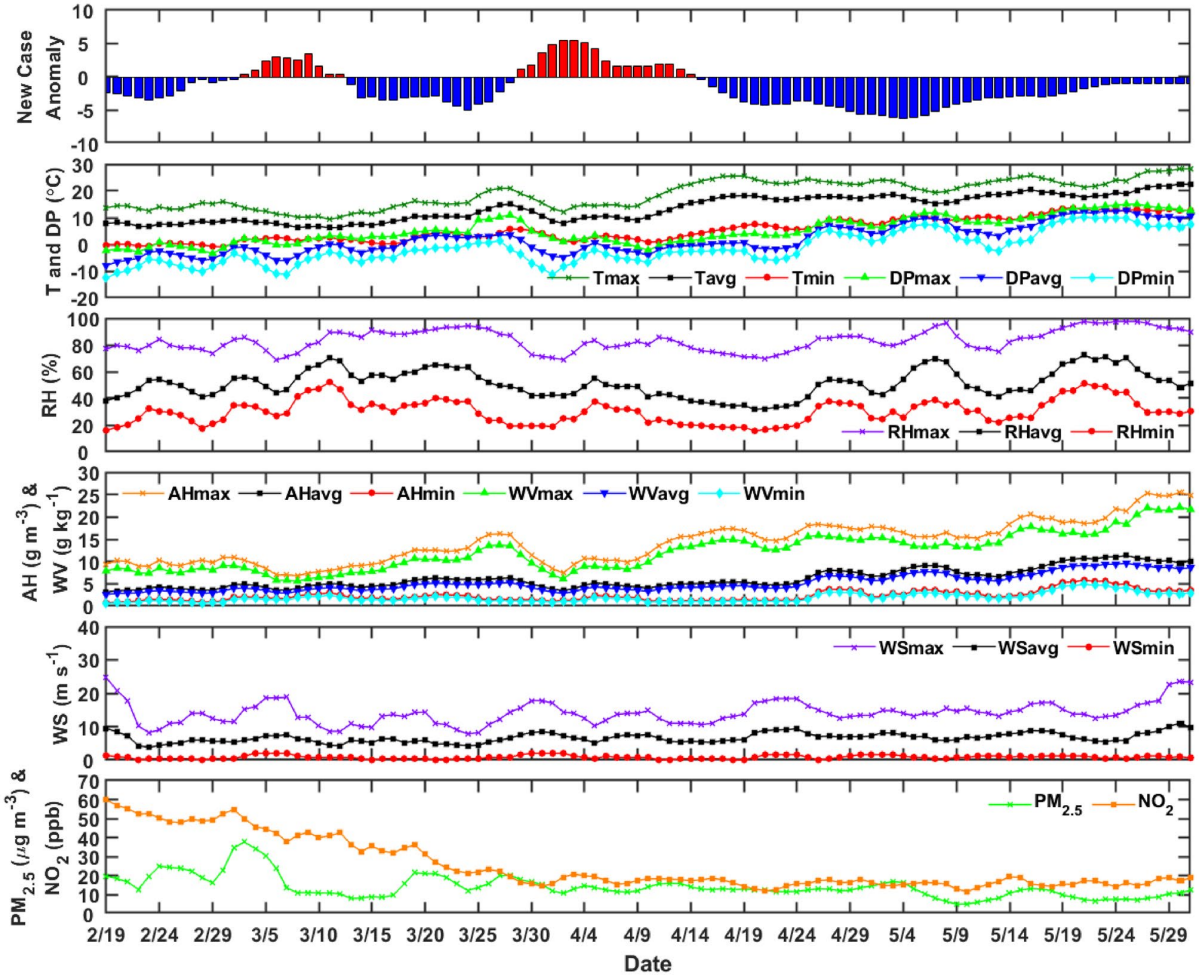
ICU patients daily case anomaly and meteorological and air pollution variables back time-shifted 20 days to take into consideration incubation and latency for the autonomous province of Trento.

As the previous two cases, Fig. 5 shows the ICU residuals and the values of the meteorological and air-quality variables in the analyzed period. Table 7 shows that the temperature has a variability similar to Milan and Florence (− 1 °C 28 °C). Instead, the Dew Point, being Trento closer to Alps, shows lower values (− 13 °C 15 °C). The relative humidity ranges from 16 to 100%, while AH and VW range from 1 to 25 g m^−3^ and 1 to 22 g kg^−1^ respectively. Those values have similar trends with respect to the other analyzed cases. The wind speed shows a similar variability (0–26 m s^−1^). Figure 5 shows a drop in PM_2.5_ and NO_2_ concentrations, more remarkable for the latter. The seasonal analysis of Table 8 likewise shows an increment in average temperature and humidity related parameters, while pressure and wind are constant. A remarkable decrease is shown in PM_2.5_ and NO_2_, with values reaching a third of February concentrations in 40 days for the latter.

**Table 7:** Descriptive statistical analyses of meteorological and air-pollution variables (Feb. 19–May 31, 2020; N = 103) in the autonomous province of Trento.

Parameters	Min	Max	Mean	SD	Median	Mode	Kurtosis	Asymmetry
T_max_ (℃)	9	28	19	5	20	23	1.7	0.0
T_avg_ (℃)	6	23	14	5	14	6	1.5	0.0
T_min_ (℃)	− 1	14	5	4	4	2	1.8	0.4
DP_max_ (℃)	− 3	15	5	5	4	2	2.0	0.3
DP_avg_ (℃)	− 8	13	2	5	0	− 1	2.1	0.4
DP_min_ (℃)	− 13	10	− 1	6	− 3	− 5	2.2	0.4
P_max_ (hPa)	979	1000	991	5	991	983	2.3	− 0.1
P_avg_ (hPa)	975	997	987	5	987	980	2.2	− 0.1
P_min_ (hPa)	973	994	984	5	985	985	2.3	− 0.1
RH_max_ (%)	69	98	83	8	83	98	2.0	0.1
RH_avg_ (%)	32	72	51	10	50	42	2.3	0.2
RH_min_ (%)	16	52	30	9	30	30	2.5	0.4
AH_max_ (g m^−3^)	7	25	14	5	15	7	2.5	0.4
AH_avg_ (g m^−3^)	3	11	6	2	5	3	2.5	0.8
AH_min_ (g m^−3^)	1	6	2	1	2	1	3.8	1.2
WV_max_ (g kg^−1^)	6	22	12	4	13	6	2.5	0.4
WV_avg_ (g kg^−1^)	3	10	5	2	4	3	2.5	0.8
WV_min_ (g kg^−1^)	1	5	2	1	2	1	3.8	1.2
WS_max_ (m s^−1^)	7.8	24.7	14.1	3.3	13.8	14.0	3.9	0.7
WS_avg_ (m s^−1^)	3.8	11.0	6.7	1.5	6.5	5.6	2.8	0.4
WS_min_ (m s^−1^)	0.0	2.0	0.9	0.6	0.8	0.4	2.5	0.5
PM_2.5_ (µg m^−3^)	5.1	37.7	14.2	6.1	12.9	5.1	6.1	1.5
NO_2_ (ppb)	11.8	60.0	25.4	13.5	18.3	15.6	2.7	1.1

**Table 8:** Meteorological parameters (mean ± SD) monthly variations in Trento, Italy for 2020.

	Feb. (N = 11)	March (N = 31)	April (N = 30)	May (N = 31)
T_avg_ (℃)	8 ± 1	9 ± 3	14 ± 4	19 ± 2
DP_avg_ (℃)	− 5 ± 2	0 ± 3	0 ± 3	9 ± 3
P_avg_ (hPa)	993 ± 4	987 ± 6	989 ± 3	984 ± 4
RH_avg_ (%)	46 ± 6	56 ± 8	43 ± 7	57 ± 10
AH_avg_ (g m^−3^)	4 ± 0	5 ± 1	5 ± 1	9 ± 2
WV_avg_ (g kg^−1^)	3 ± 0	4 ± 1	4 ± 1	8 ± 1
WS_avg_ (m s^−1^)	5.9 ± 1.8	5.9 ± 1.1	6.9 ± 1.2	7.5 ± 1.4
PM_2.5_ (µg m^−3^)	19.9 ± 3.8	17.5 ± 8.1	13 ± 1.3	10.1 ± 3.3
NO_2_ (ppb)	51.9 ± 3.9	33.5 ± 11.1	17 ± 2.1	16.1 ± 1.8

### Correlation between COVID-19 and meteorological and air-pollution variables

We investigated the correlation between the basic meteorological and air-pollution variables and COVID-19 pandemic transmission using the non-parametric Spearman and Kendall rank tests. As described in sect. 3.1, the correlation is investigated against the residual ICU hospitalized patients with a time-shift of 20 days, i.e., met and air quality data from 19 February 2020 to 30 May 2020 and ICU patients from 09 March 2020 to 19 June 2020 to take into consideration the incubation period and delay in ICU admission. We assume that hospital system did not collapse during the peak (hypothesis confirmed by the Italian health authorities). The results of non-linear correlations between COVID-19 pandemic and meteorological and air-pollution variables are summarized in Table 9 for Milan, Lombardy, in Table 10 for Florence, Tuscany and in Table 11 for the autonomous province of Trento.

**Table 9:** Spearman and Kendall non-linear rank correlation test between meteorological and air-pollution variables and ICU number patient’s residual for Milan.

Parameters	Spearman rank correlation		Kendall rank correlation	
	r_s_	p	τ	p
T_max_*	− 0.35	< 0.01	− 0.20	< 0.01
T_avg_*	− 0.36	< 0.01	− 0.20	< 0.01
T_min_*	− 0.37	< 0.01	− 0.25	< 0.01
DP_max_*	− 0.37	< 0.01	− 0.25	< 0.01
DP_avg_*	− 0.39	< 0.01	− 0.27	< 0.01
DP_min_*	− 0.39	< 0.01	− 0.26	< 0.01
P_max_*	− 0.26	< 0.01	− 0.18	< 0.01
P_avg_*	− 0.30	< 0.01	− 0.21	< 0.01
P_min_*	− 0.35	< 0.01	− 0.25	< 0.01
RH_max_**	− 0.12	0.23	− 0.06	0.35
RH_avg_**	− 0.08	0.44	− 0.04	0.59
RH_min_**	− 0.06	0.57	− 0.03	0.70
AH_max_*	− 0.38	< 0.01	− 0.23	< 0.01
AH_avg_*	− 0.40	< 0.01	− 0.27	< 0.01
AH_min_*	− 0.27	< 0.01	− 0.16^#^	< 0.05
WV_max_*	− 0.37	< 0.01	− 0.23	< 0.01
WV_avg_*	− 0.40	< 0.01	− 0.27	< 0.01
WV_min_*	− 0.28	< 0.01	− 0.16	< 0.05
WS_max_**	0.15	0.13	0.10	0.14
WSavg	0.20^#^	< 0.05	0.14^#^	< 0.05
WS_min_**	0.03	0.76	0.02	0.74
PM_2.5_*	0.27	< 0.01	0.19	< 0.01
NO_2_**	0.12	0.23	0.09	0.20

Variables with a significant statistic at 99% are indicated by *, significant at 95% by #. No correlation by **.

**Table 10:** Spearman and Kendall non-linear rank correlation test between meteorological and air-pollution variables and ICU number patient’s residual for Florence.

Parameters	Spearman rank correlation		Kendall rank correlation	
	r_s_	p	τ	p
T_max_*	− 0.28	< 0.01	− 0.19	< 0.01
T_avg_*	− 0.34	< 0.01	− 0.24	< 0.01
T_min_*	− 0.36	< 0.01	− 0.25	< 0.01
DP_max_*	− 0.41	< 0.01	− 0.27	< 0.01
DP_avg_*	− 0.35	< 0.01	− 0.25	< 0.01
DP_min_*	− 0.32	< 0.01	− 0.23	< 0.01
P_max_*	0.27	< 0.01	0.17	< 0.01
P_avg_*	0.38	< 0.01	0.26	< 0.01
P_min_*	0.36	< 0.01	0.24	< 0.01
RH_max_**	0.04	0.68	0.02	0.76
RH_avg_**	0.05	0.64	0.03	0.70
RH_min_**	− 0.03	0.76	− 0.03	0.69
AH_max_*	− 0.26	< 0.01	− 0.18	< 0.01
AH_avg_*	− 0.31	< 0.01	− 0.22	< 0.01
AH_min_*	− 0.27	< 0.01	− 0.18	< 0.01
WV_max_*	− 0.26	< 0.01	− 0.18	< 0.01
WV_avg_*	− 0.34	< 0.01	− 0.24	< 0.01
WV_min_*	− 0.35	< 0.01	− 0.25	< 0.01
WS_max_*	− 0.30	< 0.01	− 0.17^#^	< 0.05
WS_avg_*	− 0.51	< 0.01	− 0.33	< 0.01
WS_min_*	− 0.38	< 0.01	− 0.29	< 0.01
PM_2.5_*	0.38	< 0.01	0.23	< 0.01
NO_2_	− 0.23^#^	< 0.05	− 0.14^#^	< 0.05

Variables with a significant statistic at 99% are indicated by *, significant at 95% by #. No correlation by **.

**Table 11:** Spearman and Kendall non-linear rank correlation test between meteorological and air-pollution variables and ICU number patient’s residual for Trento.

Parameters	Spearman rank correlation		Kendall rank correlation	
	r_s_	p	τ	p
T_max_*	− 0.39	< 0.01	− 0.25	< 0.01
T_avg_*	− 0.36	< 0.01	− 0.22	< 0.01
T_min_*	− 0.35	< 0.01	− 0.22	< 0.01
DP_max_*	− 0.41	< 0.01	− 0.25	< 0.01
DP_avg_*	− 0.42	< 0.01	− 0.29	< 0.01
DP_min_*	− 0.44	< 0.01	− 0.32	< 0.01
P_max_**	0.10	0.32	0.06	0.34
P_avg_**	0.09	0.38	0.06	0.37
P_min_**	0.08	0.42	0.07	0.33
RH_max_	− 0.24^#^	< 0.05	− 0.15^#^	< 0.05
RH_avg_**	− 0.12	0.25	− 0.07	0.28
RH_min_**	− 0.06	0.57	− 0.03	0.65
AH_max_*	− 0.41	< 0.01	− 0.26	< 0.01
AH_avg_*	− 0.43	< 0.01	− 0.30	< 0.01
AH_min_*	− 0.27	< 0.01	− 0.17*	< 0.01
WV_max_*	− 0.41	< 0.01	− 0.26	< 0.01
WV_avg_*	− 0.44	< 0.01	− 0.30	< 0.01
WV_min_*	− 0.27	< 0.01	− 0.17*	< 0.01
WS_max_**	0.03	0.73	0.02	0.72
WS_avg_**	− 0.03	0.77	− 0.02	0.81
WS_min_**	0.13	0.20	0.09	0.23
PM_2.5_**	0.13	0.17	0.09	0.20
NO_2_*	0.31	< 0.01	0.21	< 0.01

Variables with a significant statistic at 99% are indicated by *, significant at 95% by #. No correlation by **.

Temperature, DP, AH, VW show significative negative correlation for Spearman and Kendall parameters (p < 0.01; 99% C.I) with COVID-19 pandemic transmission. These results confirm previous findings, e.g.,^4,5,19^, that virus transmission is enhanced by cold and dry climates. The wind speed does not present significant correlation. The atmospheric pressure shows a negative significant correlation, as found in^8^. On the opposite^8^, show a positive correlation with the temperature, DP and AH because the analysis is carried out without paying attention to the phase of the epidemy, i.e. in the early phase, the number of total positives will rise, then will reach a peak at maturity and then will start to descent. For this reason, without working on residuals, the analysis and results will be strongly dependent on pandemic phase. Regarding the pollutants, a positive correlation is found between PM_2.5_ concentration and cases, indicating that the pollution is facilitating the transmission. No significant correlation is found with NO_2_.

The results from Milan analysis are confirmed and corroborated in Florence (Table 10): temperature, dew point, AH and VW are negatively correlated with the COVID-19 pandemic transmission. We find a positive correlation for the atmospheric pressure, in disagreement with Milan analysis. Another substantial difference is that the wind is strongly negatively correlated, meaning that during stagnant conditions the virus easily spreads. Same results for air pollution: strong positive correlation with PM_2.5_ and not significant correlation with NO_2_.

The results in Trento (Table 11) are in strong agreement with Milan and Florence. Again, temperature, dew point, AH and WV show the strongest anti-correlation with the virus transmission. Wind is partially in agreement. PM_2.5_ is instead not significative, while NO_2_ shows a positive correlation.

In Table 12 we report the significant correlations for the analyzed cases.

**Table 12:** Spearman rank summary result showing the meteorological variables in agreement for all the three cities.

	T	DP	AH	WV	PM_2.5_
Milan	− 0.35; − 0.36; − 0.37	− 0.37; − 0.39; − 0.39	− 0.38; − 0.40; − 0.27	− 0.37; − 0.40; − 0.28	0.27
Florence	− 0.28; − 0.34; − 0.36	− 0.41; − 0.35; − 0.32	− 0.26; − 0.31; − 0.27	− 0.26; − 0.34; − 0.35	0.38
Trento	− 0.39;− 0.36;− 0.35	− 0.41; − 0.42; − 0.44	− 0.41; − 0.44; − 0.27	− 0.41; − 0.43; − 0.27	NS

PM_2.5_ is significative for Milan and Florence.

Table 12 put in evidence that T, DP, AH and WV have a strong negative correlation (comparable values for all the three analyzed cases). It is possible to speculate that cool and dry weather contribute to COVID-19 pandemic transmission. Instead, pollution as PM_2.5_ is positively correlated just for Milan and Florence. Also, the wind speed and pressure show partial correlations for some cities (sometimes discordant). For those cases, it is necessary a further analysis taking into consideration other cities to confirm or deny any possible correlation.

The main findings from other studies are reported in Table 13. To corroborate our findings, it is important to stress that lower temperatures at mid-latitudes promote indoor activities and people aggregation, facilitating the virus transmission.

**Table 13:** Main research findings on COVID-19 pandemic transmission and basic meteorological parameters.

Study	T	DP, AH, WV	Region
^8^	Positive	Positive	Singapore
^9^	Positive	N/A	China
^19^	Negative	Negative	China
^10^	No Correlation	N/A	China
^13^	Negative	N/A	Mexico
^30^	Negative	N/A	Catalunya (Spain)
^11^	Negative	N/A	China
^14^	Positive	Negative	Germany
^12^	Positive	Negative	NY City, USA

The results from this study partially confirms that air-pollution can play a role in COVID-19 pandemic, but further analysis is needed to assess if higher aerosol concentrations are able to carry the virus or just turn mild cases into severe requiring ICU hospitalization. Those results however confirm previous studies in literature that put in evidence the role of aerosol in aggravating or transmitting the SARS CoV-2 virus^15,20,31^.

This study, for the first time, investigates the correlation between basic meteorological and air-pollution variables and COVID-19 pandemic transmission not directly on the variable but on the residuals. For the analysis we used the ICU patient residuals with respect to GMM instead of the daily new positive cases variable. This approach makes the correlation independent on eventual lockdown policies and on the natural trend of viral epidemies as reported in the previous section. More research and studies are needed to assess why the COVID-19 pandemic outbreak hit stronger (also in terms of fatalities) the northern regions (Fig. 1, inset) compared to center, southern and insular regions. In contrast to^8^, this study shows limitations as the meteorological data are taken from a single observation site inside the three cities. Also, the founded correlations are specific for this temperature and humidity ranges. More studies are needed for averaged lower and higher temperatures to corroborate the outcomes provided in this paper.

## Conclusions

In March 2020, the World Health Organization declared pandemic the new COVID-19 outbreak caused by the SARS-CoV-2 virus. In this study, we investigate the correlation between the basic meteorological, air-pollution variables, and virus transmission over 103 days from 09 March 2020 to 19 June 2020 in two metropolitan areas as Milan, Lombardy and Florence, Tuscany, and the autonomous province of Trento. The first documented local transmission case dated back to 24 February 2020. To assess the correlation, differently from other studies, we considered as a reliable variable the residuals of the daily number of Intensive Care Unit (ICU) hospitalized patients with respect to the Gaussian Mixture Model (GMM). This variable, differently from others, as the. daily new positive infections, is independent from the number of performed test. Moreover, working on the residuals, makes the analysis independent on the analyzed time period. To take into consideration the incubation period and latency for admission in ICU unit, both the meteorological and air-pollution variables are 20 days back time-shifted (19 February 2020–30 May 2020). The results put in evidence that temperature, dew point temperature, absolute humidity, water vapor are negatively correlated with the virus transmission. Those findings confirm other studies for mid-latitude regions. Wind speed and atmospheric pressure show a certain degree of correlation but the results not unanimous. For this reason, more analysis is needed in other cities also in other geographical regions. The PM_2.5_ concentration positively correlates with SARS CoV-2 transmission. From those results, it is possible to speculate that air-conditioned environments not using sub-micron filters for organic particulate might help the virus transmission.

The results from this analysis suggest that further studies are needed to investigate why in some parts of Italy, and more in general, of the world, the virus transmission is different. This methodology that extracts information from the residuals can help to quantitatively establish if the differences in meteorological and air-pollution variables played a role in flagging the virus transmission in different Italian metropolitan areas spared by the virus. Those results can promote further studies in other parts of the world testing also other air-pollution related variables, e.g. Carbon Monoxide, Sulfuric dioxide, tropospheric ozone. It is also important to stress that both the meteorological and air-pollution variables are co-factors in COVID-19 pandemic transmission. Their influence is still marginal while all the epidemiological aspects should not be neglected and have obviously the primary role.
